# The Proclivities

**Published:** 1856-01

**Authors:** 


### THE PROCLIVITIES

Of the age are towards every man becoming his own shoe-black. Newspaperdom is getting wise, very. Every editor who can boast of a circulation of fifteen, dead heads and exchanges not included, sets himself up as Sir Oracle on every subject under the sun—some even going higher, not a few daring to interpret the meaning of Scripture for the guidance of their readers, and meeting with no resistance, they have made bold to invade the time-honored domain of us Doctors. Now, having some considerable affinity for *Friends'* principles, we content ourselves with a decided protest, not however wishing it to be understood to guarantee a literal adherence, or that we might not "bolt" if provocations are intensified.

We have no doubt that the world would be the better for it, if every newspaper in the land were to rigidly exclude from its columns everything which had a bearing on health, except such as referred to the daily habits of life, and even these to admit with caution, knowing, as we do, that many things which, at the first glance appear rational and useful, will not bear the light of investigation.

The N. Y. Observer for Nov 27 informs its readers that *teeth are rendered insensible to pain* by inserting a pill into the hollow of the tooth made of Canada Balsam and slacked lime, and that immediate relief is afforded in *all* tooth-aches but those arising from chronic inflammation—closing with the usual twaddle about its being 'SAFE, SIMPLE, and can be easily tried by any person.' Suppose this were so, how many people know what *Canada Balsam* is, or where to get it? Certainly it would require less time to consult an intelligent dentist or physician, with the advantage of being on the safe side, for if it did arise from 'chronic inflammation,' from a sac at the root, the pill, by hardening and closing the natural vent, would aggravate the pain to an extent unendurable, or force a fistulous ulcer in some part of the jaw. Our advice is, if any thing is the matter with your teeth, go to a good dentist at once, and even if nothing is the matter, consult him twice a year, and compel each one of your children to do the same, from the age of five years up to the time of marriage, and these children will have reason to thank you for it to the close of life.

